# High Intake of Energy and Fat in Southwest Chinese Women with PCOS: A Population-Based Case-Control Study

**DOI:** 10.1371/journal.pone.0127094

**Published:** 2015-05-20

**Authors:** Jing Zhang, Ying Liu, Xiaofang Liu, Liangzhi Xu, Lingling Zhou, Liulin Tang, Jing Zhuang, Wenqi Guo, Rong Hu

**Affiliations:** 1 Department of Obstetrics and Gynecology, West China Second University Hospital, Sichuan University, Chengdu, Sichuan, People’s Republic of China; 2 Department of Ultrasound, West China Second University Hospital, Sichuan University, Chengdu, Sichuan, People’s Republic of China; 3 Clinical Laboratory center, West China Second University Hospital, Sichuan University, Chengdu, Sichuan, People’s Republic of China; Peking University Third Hospital, CHINA

## Abstract

**Background:**

Polycystic ovary syndrome (PCOS) is a common reproductive endocrinological disease with heterogeneous phenotype. Obesity contributes to the increased prevalence and severity of PCOS. Whether the intakes of major nutrients are higher in Chinese PCOS patients is still unknown.

**Objectives:**

To study the intakes of total energy, protein, fat and carbohydrate in Southwest Chinese PCOS patients.

**Methods:**

1854 women were included in the cross-sectional study. A population-based case-control study was conducted. The dietary habits and nutrients intake status of 169 PCOS patients and 338 age-matched controls were investigated by the method of semi-quantitative food frequency questionnaire.

**Results:**

The actual intake of total energy (*P* = 0.01) and fat (*P* = 0.01) were higher, but carbohydrate was lower (*P* = 0.01) in PCOS patients as compared with the controls. The energy percentage supplied by protein (12.33%±2.27% vs. 19.26%±5.91%, *P<0*.*001*) and carbohydrate (48.72%±6.41% vs. 68.31%±8.37%, *P<0*.*001*) were lower in Southwest Chinese PCOS patients than those of control, however, the energy percentage supplied by fat was higher (38.95%±5.71% vs. 12.42%±5.13%, *P<0*.*001*) in PCOS.

**Conclusions:**

Limit the intake of total energy and fat shall be recommended to the Southwest Chinese PCOS patients. Women with PCOS in Southwest China shall consult with the nutritionist for improving the dietary structure.

## Introduction

Polycystic ovary syndrome (PCOS) is a common but complex reproductive endocrinology disease, affecting both the adolescent and reproductive women. It involves reproductive system, endocrinologic system, metabolic system, gynecological cancer and obstetric events, which are characterized by irregular menstrual cycle, infertility, hirsutism, acne, obesity, insulin resistance, diabetes mellitus, dyslipidemia, endometrium carcinoma, gestational diabetes mellitus and preterm delivery [1]. PCOS may occur from the adolescence, and continues to the menopause, having a strong impact on the life quality of the whole life and the population quality of the offspring.

Obesity is a prevalent characteristic of PCOS, with a pooled estimated prevalence of 49% [2]. Abdominal adiposity, obesity, and insulin resistance (IR) are involved in the pathogenesis of PCOS. There may be a bidirectional interaction between PCOS and weight, with PCOS driving weight gain and weight gain contributing to an increased prevalence and severity of PCOS [3]. Obesity may deteriorate the severity of menstrual irregularity and insulin resistance, and may have significant impact on the long-term health risks and reproduction function of PCOS women. Progressive worsening of insulin resistance with age is primarily driven by the increase of body mass index (BMI) and may contribute to the higher risk of type 2 diabetes mellitus (T2DM) in women with PCOS [4].

Given the association between obesity, insulin resistance and the presentation of PCOS, weight management is a key initial treatment strategy for PCOS which can improve the reproductive, metabolic and psychological performance [1]. Nutritional and dietary factors are speculated to be the risk factors for PCOS. Women with PCOS may additionally have increased intake of high glycemic index (GI) foods [5] and reduced physical activity [6]. However, no difference in energy, dietary intake, physical activity or muscle strength is found between lean or overweight women with or without PCOS in some studies [5, 7–8]. The BMI varies considerably across countries for women with PCOS. There is a lower BMI in Chinese women with PCOS as compared with Caucasian population [9]. The dietary composition varies among population with different race and territory. The dietary situation of Chinese PCOS patients is unclear. Whether the intakes of major nutrients supplying energy are higher in Chinese PCOS patients is still unknown.

The intakes of total energy, protein, fat and carbohydrates were investigated between Chinese women with PCOS and control women in present study to clarify the relationship among them.

## Methods

### Study design

A population-based case-control study was carried out in Chengdu, Sichuan. The study protocol of the cross-sectional survey has already been partly published in our previous article including sample size calculation, random cluster sampling, participants’ selection, questionnaire investigation, ultrasound examination, blood collection and experimental tests [10]. Sample size calculation was based on the primary outcome of the prior study assessing the prevalence of PCOS among women in Chengdu. Because the prevalence of PCOS differed greatly in different age groups and regions, we chose a moderate prevalence (P_0_ = 0.04) for sample size calculation. The calculated sample size was 1475. Considering a dropout rate of 10%, our final sample size was 1623. The age constituent ratio of women obtained from the population census in the 2004 Yearbook of Sichuan Province was used to calculate the number of participants needed in each age group. Five communities, two universities and three middle schools from nine districts of Chengdu city were randomly cluster sampled to collect the participants.

The PCOS patients identified from the cross-sectional study were collected as the case group in the case-control study. The control number was calculated based on the number of PCOS patients with the ratio of 1:2 in each age group, respectively.

The study was approved by the Human Ethics Committee of West China Second University Hospital. Information and consent forms were obtained from all the subjects or their guardians.

### Participants

1854 women, aged 12–44 years, who have had menses for at least 2 years, who have lived in Chengdu for at least 6 months, and who agree to answer the questionnaires were included in the cross-sectional study. Participants who had used hormones or drugs which could apparently affect endocrinological function in the previous three months were excluded. Participants with the history of diabetes mellitus, hypertension, heart disease, cerebrovascular disease, liver and kidney diseases, cancer, organ transplantation, nutrition-related diseases were excluded, pregnant or lactation women were also excluded.

The diagnosis of PCOS was based on the Rotterdam criteria, with the association of at least two of the three following criteria [11]. Cushing’s syndrome, thyroid disease, androgen-secreting tumors, congenital adrenal hyperplasia and prolactinoma were excluded by further measuring the plasma levels of prolactin, thyroid stimulating hormone (TSH), free triiodothyronine (FT 3), free thyroxine (FT 4), testosterone and plasma total cortisol (PTC). Controls matched for sampling cluster and age, were randomly chosen from the non-PCOS participants of cross-sectional investigation with the ratio of 1:2.

### Measurements

Anthroposomatology indexes including height, body weight, body mass index, hip circumference, waist circumference, waist hip ratio (WHR), systolic and diastolic blood pressure were measured; meanwhile acne, hirsutism, baldness and acanthosis nigricans were evaluated individually by two trained investigators.

Overnight fasting blood samples (15 ml) were collected from all the participants. Hormonal and metabolic assessments were made between the 2nd and 10th days of the menstrual cycle or on any day if the patient was amenorrhea. The plasma was separated and stored at *−*20°C until assay. Estradiol (E_2_), testosterone (T), free testosterone (FT), progesterone (P), follicle-stimulating hormone (FSH), luteinizing hormone (LH), fasting insulin (FINS) and fasting plasma glucose (FPG) were measured by chemiluminescence method, glucose oxidase method and radioimmunoassay, respectively, with inter- and intra-assay coefficients of variation of <15% and <10%. Serum FT was measured using radioimmunoassay method (Diagnostic Products Corp., Los Angeles, Calif., USA), FINS and T were measured using chemiluminescence method (Siemens Medical Solutions Diagnostics Limited.) All of above tests were performed by professional laboratorian in the clinical test center of our hospital. It had been accredited by ISO 15189 in 2006. At the same day, transvaginal or transabdominal ultrasound was examined by a professional doctor to measure the endometrium and ovaries. Ultrasound criteria for PCO: in the early follicular phase, one or both ovaries with the number of small follicles (diameter of 2–9 mm) ≥ 12, and/or ovarian volume ≥ 10 ml. Ovarian volume was calculated according to a simplified formula for an ellipsoid (0.5×length×width×thickness).

Participants with fasting glucose >6.1 mmol/L and fasting insulin >15 mU/L were defined as hyperglycemia and hyperinsulinemia respectively [12]. Participants with BMI≥28 kg/m^2^ or WHR≥0.8 were classified to be obesity or abdominal obesity in Chinese, respectively [13]. Insulin resistance was estimated by homeostasis model assessment (HOMA). HOMA index was calculated by multiplying insulin (mIU/mL) by glucose (mmol/L) and dividing the product by 22.5, and HOMA value >2.77 was classified as insulin resistance [14].

### Questionnaire Survey

General situation, childbearing history, menstrual history, family history, psychological state, medical conditions and dietary habits were investigated. The survey was conducted by using the combination of face-to-face interview and telephone inquiries. If the face-to-face interview was unavailable, telephone interview was applied as the supplemental method, and detailed records were taken. The participants were ought to be surveyed by face-to-face interview at the first time, if the content of the questionnaire had to be further clarified, then telephone interview could be applied. Investigators received a 1-day on-site training, and they could take part in the survey after passing the examination. Pre-survey among investigators was carried out before the formal investigation to ensure that they have known the communication skill. All of the participants were ensured to get an integral and clear questionnaire. Quality control of the investigation was carried out by the inspectors who were in charge of checking the data of each questionnaire. Two staffs input the data independently using the Epidata3.1 software and would check the data again before inputting. If inconsistencies were found, the questionnaires were checked and the participants were phoned immediately for clarification.

Semi-quantitative food frequency questionnaire (SQFFQ) could effectively reflect the dietary habits and nutrients intake status of the target population, and was an economical and reliable method in the large sample population surveys [15, 16]. The revised SQFFQ used in present study was developed from a previous SQFFQ in Chongqing, China [17]. Women were asked how often, on average, they had consumed each type item during the previous year. Standard units or serving sizes were specified for each food item in the frequency food questionnaire, which had nine possible responses for each item, ranging from ‘never or once per month’ to ‘four or more times per day’. Average consumption of food was defined as the multiples (0, 0.5, 0.8, 1, 1.5, 2, 3 or more) of the reference food weight. Reference food weight referred to the average unit of food weight in grams. For instance, standard units for rice, pumpkin, sausage and milk were defined as 100 g, 150 g, 30 g and 200 g, respectively. Colored pictures of the reference food were used to help the recall. The calculation of nutrients intake was based on the 2002 Chinese Food Composition Table [18]. The daily intake of nutrients was calculated with the average daily food weight multiplying by average nutrients composition contained in each food. The reliability and validity of the revised SQFFQ including 18 categories and 120 kinds of food, has been found eligible for investigating the role of nutrients in disease development in Chengdu female residents, especially for investigating the relationship of metabolism and diseases [19]. Food categories included frumentum, bean products, fresh beans, rhizomatic vegetables, melons, leaf vegetables, fruits, nuts, livestock meat, poultry meat, milk, eggs, aquatic products, fungus, salted vegetables, drink, nutritious supplementary, edible oil and condiment. 120 different food items which were frequently consumed in Sichuan province were surveyed. Sausage and bacon made from pork were the traditional foods in this district and were popular with most residents. The validity was assessed by comparing the SQFFQ with the "standard" method of 3 days dietary recall, and the reliability was tested by comparing the first SQFFQ with the second SQFFQ at four weeks’ interval. For reliability, the average correlation coefficient (CC) was 0.66 and reduced to 0.60 after adjusting for energy, the average of intra-class correlation coefficients (ICC) was 0.65. For validity, the average CC was 0.35 and remained stable after adjusting for energy or nutrients’ CC.

### Statistical analysis

Results were expressed as mean±SD or median and interquartile range (IQR) depending on their normal or skewed distribution. Comparisons between the two groups were analyzed by Student’s t-test or Mann-Whitney U test. Mann-Whitney U test was only used for the comparison of actual nutrient intake including energy, protein, fat and carbohydrate. Comparisons between ratios were carried out with the use of the Chi-square test. Multivariate logistic regression analyses were used to determine the independent effect of dietary nutrients with PCOS as dependent variable. All analyses were performed with the Statistical Package for the Social Sciences, version 16 (SPSS). Data from SQFFQ was entered in duplicate in Epidata software, version 3.1 (Epidata Association) and subsequently transported to SPSS for analysis. Data was considered to be significant at *P*<0.05

## Results

169 PCOS patients, 338 age and cluster matched control participants were enrolled in present case-control study. The baseline characteristics including age (22.07±6.10 years vs. 22.08±6.09 years, *P* = 0.979), BMI (20.56±2.65 kg/m^2^ vs. 20.07±4.28 kg/m^2^, *P* = 0.175), WHR (0.78±0.05 vs. 0.79±0.43, *P = 0*.*800*), systolic blood pressure (104.38±10.65 mmHg vs. 100.48±12.16 mmHg, *P = 0*.*536*), diastolic blood pressure (67.6±10.5 mmHg vs. 66.37±9.90 mmHg, *P = 0*.*197*), ln HOMA (0.76±0.66 vs. 0.72±0.52, *P = 0*.*468*), and age of menarche (12.76±1.25 years vs. 12.84±1.41 years, *P = 0*.*541*) were not different between participant with PCOS and controls. The birth weight was lower (2.85±1.07 kg vs. 3.02±0.81 kg, *P* = 0.049) in PCOS patients than those of controls. The clinical characteristics between the groups were showed in Table 1.

**Table 1:** The clinical characteristics between PCOS and control participants.

	PCOS^1^	Control	*P* ^*#*^
	n(%)	n(%)	
Oligo-anovulation	160 (94.7)	117 (34.6)	0.001*
Hirsutism	68 (40.2)	38 (11.2)	0.001*
Acne	70 (41.4)	120 (35.5)	0.194
Unilateral PCO^2^	69 (40.8)	16 (4.7)	0.001*
Bilateral PCO	39 (23.1)	1 (0.3)	0.001*
BMI^3^ ≥ 28 (kg/m^2^)	16 (16.6)	7 (2.1)	0.001*
WHR^4^ ≥ 0.8	48 (28.4)	78 (23.1)	0.191
Hyperglycemia	81 (47.9)	84 (24.9)	0.001*
Hyperinsulinemia	20 (11.8)	25 (7.4)	0.098
IR^5^	60 (35.5)	82 (24.3)	0.008*
Diabetic family history	20 (11.8)	12 (3.6)	0.001*
Psychiatric family history	2 (1.2)	3 (0.9)	0.751
Epilepsy family history	3 (1.8)	4 (1.2)	0.346
Baldness family history	39 (23.1)	31 (9.2)	0.001*

^1^ PCOS, polycystic ovary syndrome;

^2^ PCO, polycystic ovary;

^3^ BMI, body mass index;

^4^ WHR, waist hip ratio;

^5^ IR, insulin resistance;

* *P*<0.05

^*#*^ Chi-square test

The prevalence of obesity, hyperglycemia, IR, diabetic and baldness family history in PCOS group were higher than those in the control group, however, the abdominal obesity (WHR≥0.8), hyperinsulinemia, psychiatric and epilepsy family history were not significantly different between groups (Table 1). The prevalence of diabetes among the first and secondary relatives of PCOS patients was 11.83%, and 3.55% for the control group (*P = 0*.*01*). The prevalence of baldness among the male relatives of PCOS patients was 23.08%, and 9.17% for control group (*P = 0*.*01*). Logistic regression analysis showed that the family history of diabetes (*P* = 0.037, *OR* = 80.91) and baldness (*P* = 0.006, *OR* = 108.51) were related to the occurrence of PCOS.

As compared with the control group, increased levels of FT, T, LH, FPG were found in the PCOS patients, but with decreased level of FSH. No differences were found in the levels of E_2_ and FINS (Table 2).

**Table 2:** Hormonal and metabolic results of PCOS and control participants.

	PCOS^1^	Control	*P* ^#^
	(n = 169)	(n = 338)	
FT^2^ (pmol/L)	10.4 ± 3.1	8.0 ± 2.3	0.01*
T^3^ (nmol/L)	242.9 ± 83.3	201.3 ± 65.9	0.01*
E_2_ ^4^ (pmol/L)	179.6 ± 91.5	177.0 ± 98.3	0.32
LH^5^ (IU/L)	7.9 ± 4.6	5.7 ± 3.3	0.01*
FSH^6^ (IU/L)	5.2 ± 2.1	5.8 ± 2.5	0.01*
FINS^7^ (pmol/L)	61361.7 ± 28486.9	57670.2 ± 29322.7	0.97
FPG^8^ (mmol/L)	6.0± 0.9	5.8 ± 0.5	0.01*

^1^ PCOS, polycystic ovary syndrome;

^2^ FT, free testosterone;

^3^ T, testosterone;

^4^ E_2_, estradiol;

^5^ LH, luteinizing hormone;

^6^ FSH, follicle stimulating hormone;

^7^ FINS, fasting insulin;

^8^ FPG, fasting plasma glucose;

* *P* < 0.05

^#^ Student’s t-test

The actual intake of total energy (*P* = 0.01) and fat (*P* = 0.01) in Southwest Chinese PCOS patients were higher than those in control group (Table 3). The protein intake was not different between the groups (*P* = 0.23). However, decreased level of carbohydrate intake was found in PCOS group (*P* = 0.01). Median and interquartile range were used to express the intake of energy and macronutrients for the skewed distribution.

**Table 3:** Comparison of actual nutrient intake between PCOS and control participants.

Nutrients	PCOS	Control	Z^※^	*P*
	Median (IQR^1^)	Median (IQR)		
Energy (KJ)	10837.2 (9854.3, 11833.8)	7173.1 (5894.8, 8033.9)	−16.4	0.01*
Protein (g)	66.3 (58.9, 74.4)	67.4 (58.7, 79.7)	−1.2	0.23
Fat (g)	95.8 (88.1, 99.7)	90.58 (74.8, 97.8)	−4.8	0.01*
Carbohydrate (g)	231.3 (193.4, 261.9)	336.5 (282.7, 402.2)	−10.1	0.01*

^1^ IQR, interquartile range

* *P* < 0.05

^※^ Mann-Whitney U test

The energy percentage supplied by protein (12.33%±2.27% vs. 19.26%±5.91%, *P<*0.001) and carbohydrate (48.72%±6.41% vs. 68.31%±8.37%, *P<*0.001) were lower in southwest Chinese PCOS patients than those of controls, however, the energy percentage supplied by fat was higher (38.95%±5.71% vs. 12.42%±5.13%, *P<*0.001). Carbohydrate was still the first major nutrient supplying energy for both the PCOS and control, but the ratios for the PCOS patients were lower than the normal level, which was recommended to be 70%. The energy percentage provided by fat for PCOS patients was higher than the recommended level of 25%. The energy percentage supplied by the macronutrients for the PCOS patients in Southwest China were diverged from the levels recommended by the nutritionist.

Intakes of fat, carbohydrate, cholesterol, diet fiber, riboflavin, niacin, vitamin E, calcium, magnesium, ferrum as gram, and total energy intake as kilo joule (KJ) were analyzed to assess their roles in the incidence of PCOS. BMI was chosen to be the adjusted factor while analyzing, but confounding influence of BMI was not found. Higher intake of total energy (*B* = 0.002, *P* = 0.001, *Exp(B)* = 1.002, *95%CI* 1.001, 1.002), higher intake of fat (*B* = 0.022, *P* = 0.01, *Exp(B)* = 1.022, *95%CI* 1.005, 1.039) and lower intake of carbohydrate (*B* = 0.005, *P* = 0.017, *Exp(B)* = 1.004, *95%CI* 1.001, 1.007) were found to be related to the incidence of PCOS.

Top 20 food items were found to be different for supplying fat, carbohydrate and protein between PCOS and control subjects respectively, which were listed in Table 4. Seven different food items were consumed as the top three food categories for supplying different macronutrients, and were chosen to be statistically compared between PCOS and control using Mann-Whitney U test. The actual intake amount of rice (Z = -0.645, *P =* 0.519), apple (Z = -0.988, *P =* 0.323), pork (Z = -1.651, *P =* 0.099), lean hogs (Z = -0.488, *P =* 0.626), colza oil (Z = -0.068, *P =* 0.946), and soy bean sauce (Z = -0.417, *P =* 0.676) were not different between PCOS and control, except for the noodle (Z = -2.061, *P =* 0.039).

**Table 4:** Top 20 food items consumed by participants for supplying fat, carbohydrate and protein.

PCOS						Control					
Food type	Fat intake amount (g/d)	Food type	Carbohydrate intake amount (g/d)	Food type	Protein intake amount (g/d)	Food type	Fat intake amount (g/d)	Food type	Carbohydrate intake amount (g/d)	Food type	Protein intake amount (g/d)
Colza oil	899.1	Rice	123.2	Rice	12.0	Colza oil	865.8	Rice	115.5	Rice	11.3
Pork	18.5	Soy bean sauce	6.3	Lean hogs	9.8	Pork	18.5	Noodle	7.8	Lean hogs	9.8
Lean hogs	4.0	Apple	4.3	Pork	6.6	Lean hogs	4.0	Soy bean sauce	6.1	Pork	6.6
Milk	3.2	Noodle	3.7	Ajinomoto	4.0	Milk	3.2	Apple	4.3	Soybean sauce	3.4
Rice	2.9	Flour	3.5	Soy bean sauce	3.5	Rice	2.7	Flour	3.5	Milk	3.0
Bacon	1.7	Milk	3.4	Milk	3.0	Chili sauce	2.3	Milk	3.4	Noodle	1.4
Chili sauce	1.4	Bread	3.1	Egg	1.3	Bacon	1.7	Bread	3.1	Egg	1.3
Egg	1.1	Orange	3.0	Flour	0.8	Egg	1.1	Orange	3.0	Flour	0.8
Instant noodle	0.8	Steamed bun	2.7	Noodle	0.7	Sunflower seed	1.0	Chili sauce	2.7	Chili sauce	0.7
Sunflower seed	0.6	Ajinomoto	2.7	Pig's feet	0.6	Peanut	0.7	Steamed bun	2.5	Sunflower seed	0.6
Pork side ribs	0.6	Instant noodle	2.5	Chicken	0.5	Pork side ribs	0.6	Banana	2.2	Pig’s feet	0.6

## Discussion

The dietary status in adolescent and reproductive-age Southwest Chinese PCOS patients was explored in present population-based case-control study for the first time. Increased intake of total energy and fat, decreased intake of carbohydrate were found in PCOS patients in present study. The energy percentage provided by fat was higher, but carbohydrate was lower for Southwest Chinese PCOS patients. High intake of total energy and fat, low intake of carbohydrate were correlated with the PCOS. No difference was found for protein intake between PCOS and control in present study. The nutrients components for the PCOS patients in Southwest China were found to be diverged from the levels recommended by the nutritionist.

### Nutrients intake of participants

The nutrients intake of PCOS patients have been previously studied in Caucasian population. Increased actual fat intake but low intake of carbohydrate was also found by two studies [20, 21]. Wild et al. found an increased fat intake and a reduced fiber intake in patients with PCOS compared with controls, yet this difference was actually related to difference in weight because the patients were much heavier than the controls [20]. Dietary intake, glycemic index and glycemic load measured by a 7d food diary were compared in thirty-eight PCOS and twenty-eight age- and weight-matched controls (mean age 30.2±6.1 years) [21]. Both lean and obese participants were included in this study. However, whether the BMI was different between groups wasn’t reported. Percentage energy from carbohydrate intake was significantly lower (41%±6% vs. 48%±5%, *P*<0.001) and percentage energy from fat significantly higher (40%±6% vs. 34%±5%, *P* = 0.002) for lean PCOS than lean controls, which was in accordance with the results of our study. No difference was found on the percentage energy from cholesterol, fat and protein for obese participants. PCOS participants were not meeting dietary recommendations for fat or carbohydrate intake in this study, which was accordance to the results of our study.

However, no significant differences were found in the actual intakes of protein, fat and carbohydrate between PCOS and control in the other Caucasian studies [5, 7, 22–25]. The dietary intake of 30 women with PCOS and 27 control women were collected from a food questionnaire and a 4-day food record [5]. The age (28.9±6.5 years vs. 28.9±6.3 years, *P* = 0.96) and BMI (29.1±4.8 years vs. 29.7±4.8 years, *P* = 0.68) were comparable between groups. The PCOS group consumed 49.5% energy from carbohydrate and 34.8% energy from fat. The control group consumed 52.9% energy from carbohydrate and 31.0% energy from fat. The PCOS group consumed less carbohydrate (*P =* 0.33) and more total fat (*P =* 0.22) than did control-group women, but these differences were not statistically significant. Percentage energy from carbohydrate intake was lower but percentage energy from fat was higher in PCOS patients, which was accordance with the results of our study. The PCOS group consumed significantly more white bread and tended to consume more fried potatoes. The reliability and validity of dietary questionnaire used in this study were unclear, and the investigated duration of dietary status was not reported. The dietary situation in the previous six months was surveyed by Wright et al. for 40–50 years old PCOS patients and controls using block food frequency questionnaire with good reliability and validity [7]. Overall comparison of overweight and obese women with and without PCOS showed no significant difference in actual dietary intake, whereas lean patients with PCOS showed reduced intake of total caloric (1398.53±336.68 kcal/d vs. 1792.74±385.15 kcal/d, *P*<0.001), carbohydrates (*P* = 0.004), protein (*P* = 0.001) and fat (*P* = 0.003). However, the limitation of the survey was hospital-based and the participants were perimenopausal. Dietary intakes in 10 hypothalamic amenorrhea women and 8 PCOS patients with the age of 18–35 years were surveyed by means of a food frequency questionnaire and a seven-day food diary [22]. The BMI of PCOS patients was much higher (24.3±3.5 kg/m^2^ vs. 19.9±2.3 kg/m^2^, *P* = 0.006). A similar macronutrient distribution for women with PCOS or hypothalamic amenorrhea was found (about 16% protein, 33% fat, 52% carbohydrates). However, the comparison between PCOS patients and normal controls was not performed in this study. Diet composition of overweight or obese premenopausal women reporting weight control was estimated by a modification of the semi-quantitative Harvard Service Food Frequency Questionnaire [23]. Participants in the control group were younger than PCOS patients (26.3±7.6 years vs. 32.2±7.5 years, P = 0.003), but without difference for BMI between groups. The total caloric intake (2374±681 kcal/day vs. 2368±702 kcal/day, *P* = 0.869), proportion of fat (37%), carbohydrate (46%) and proteins (18%) in PCOS patients were not different from controls. Both groups of women maintained higher fat intakes than those currently recommended. Lean participants weren’t included in this study, and the confounding influence of weight management couldn’t be ruled out. PCOS patients and controls with 14–35 years old were investigated for the dietary intake by using a food frequency questionnaire previously validated [24]. The prevalence of obesity was 44.3% in PCOS women and 31.8% in control women (*P* = 0.288). The calorie (*P = 0*.*024*) consumption of PCOS women was greater than that of the control group regardless of age and BMI, but without differences among the intake of carbohydrate (52%), fat (25%) and protein (15.5%). Obese or overweight (BMI >25 kg/m^2^) women with PCOS and control were investigated by means of the 7 days food diary. Diet did not differ between the two groups regarding energy, protein, fat and carbohydrate, but PCOS individuals had higher consumption of high-GI foods [25]. In general, low percentage energy from carbohydrate intake but high percentage energy from fat were found in most Caucasian PCOS patients as compared with the recommended levels.

Only one study was found to investigate the dietary intake of Asian PCOS patients (age 25–40 years) and infertility controls using a 3-d dietary record [26]. Taiwanese women with PCOS consumed lower energy (6311±1408 KJ/d vs. 6766±1080 KJ/d, *P* = 0.002) and carbohydrate (51.5%±8.9% vs. 55%±6%, *P<*0.0001), but higher fat intake (30.8%±7.9% vs. 28.3%±5.1%, *P<*0.0003) compared with those with non–PCOS-related infertility. However, no difference was found for the intake of protein (17.3% ±7.3% vs. 17.6%±4.2%, *P<*0.0001) between groups. BMI (23±4.4 kg/m^2^ vs. 21.3±2.9 kg/m^2^, *P* = 0 033) was significantly higher in the PCOS group than that in the control group. The results of percentage energy were accordance with those of our study. However, the reliability and validity of dietary questionnaire used in this study were unclear, and the dietary intake between PCOS and normal participants were not compared in this study.

The limitation of the above previous studies were hospital-based case-control study, the reliability and validity of dietary questionnaire were unclear, the investigated duration of dietary status was not reported, using hypothalamic amenorrhea women or infertility patients as controls, respectively. No study explored the dietary status of Southwest Chinese PCOS patients with population-based case-control study in previous years.

The results of above studies exploring the dietary status of PCOS patients are contradictory. These data should be interpreted with caution, because of different dietary habits as well as ethnic origins among different countries, which could account for the differences among the studies. The difference might also be induced by the different proportions of overweight and obese women with PCOS in included participants.

Nowadays, with the widespread application of the weight management for PCOS, a better dietary intake was reported to be applied by PCOS patients, as reflected by improved diet quality, lower saturated fat and glycemic index intake and higher fiber and micronutrient intake, but a higher energy intake and increased amount of sedentary time compared with controls [27].

### Biochemical and clinical characteristics of participants

The present study demonstrated that women with PCOS had much more proportion of oligo-anovulation, hirsutism and polycystic ovary, which was consistent with clinical characteristics of PCOS. The prevalence of oligo-anovulation, hyperglycemia, hirsutism, acne, polycystic ovary, obesity and IR were 94.67%, 47.93%, 40.24%, 41.42%, 63.91%, 16.57% and 35.5%, respectively. The BMI of PCOS patients in present study was similar to the BMI (22.2±4.2 kg/m^2^) reported by a large sample size cross-sectional study conducted across China [28]. Li et al. reported higher prevalence of hyperandrogenism (85%), PCO (81%) and lower prevalence of insulin resistance (14.2%) than those in present study [29]. Zhang et al. also reported higher PCO (92.4%), obesity (36.6%), but lower IR (28.2%), oligo-anovulation (86.6%), hyperandrogenism (24.1%), hirsutism (8.1%), acne (15.6%) in northeast China than those in present study [29]. The difference of the phenotype might be induced by different age-group women included in the studies. Both adolescent girls and reproductive age women were included in present study, but only reproductive age women in the other studies.

The prevalence of oligo-anovulation, obesity, insulin resistance in present PCOS patients was similar to the results of previous Asian studies, but with lower obesity than Caucasia [30, 31]. However, the prevalence of hirsutism and acne were higher than Japanese but lower than Caucasian. These might be induced by the different races and dietary habits. In present population-based case-control study, the prevalence of polycystic ovary was lower than the results of hospital-based case-control study.

The present study demonstrated that women with PCOS had increased serum free testosterone, testosterone, LH, fasting plasma glucose levels and decreased FSH, which were consistent with hormonal characteristics of PCOS. However, the serum insulin level was not significant different between groups. As expected, diabetic family history and bald family history were higher in PCOS patients. The history of diabetes and baldness in the immediate family might be the risk factors of PCOS for women, which was in accordance with previous studies [32].

### The strengths and limitations of the present study

The strengths of this study include the population-based nature of the sample which minimizes the selection bias. We selected 12–44 years old female population at each age group according to the Sichuan yearbook, which made it more representative of the real status of Chengdu females. This study provided us a reliable tool in the research of the relationship between disease and dietary habits for Southwest Chinese women.

In present study, we used the standard-weight pictures of food (per bowl, dish, etc.) and colored food atlas to help the subjects recall, making the estimation of the food more objective and more realistic. The study was based on the actual eating habits of the female residents in Chengdu.

All the investigations were carried out by well-trained and experienced investigators, making most of the interviews processed successfully. In addition, without the restrictions of time and traffic, telephone interviews greatly improved the acceptance and compliance of the subjects.

However, our study had a few limitations. The 2002 China Food Composition Table failed to include all foods took by Southwest Chinese women in the questionnaire, so an objective calculation based on that table might affect some nutrient intake results. Personal memory is influenced by the educational background, physical condition, gender and mood. The participants’ recall bias was an important factor that affected the results in our investigation. An important limitation of our study was that it was a cross sectional investigation of the association of dietary factors with the PCOS. Prospective cohort study isn’t conducted; it is difficult to draw the definite conclusions about the effect of diet in this population. Until large prospective studies firmly establish the effects of diet on PCOS features, cross-sectional analyses like the present report might provide potentially useful insights in the association between diet and PCOS characteristics.

## Conclusions

Limitation the intake of total energy and fat shall be recommended to the Southwest Chinese PCOS patients. Women with PCOS in Southwest China shall consult with the nutritionist for improving the dietary components. Cohort study excluding the influence of obesity shall be carried out to explore the relationship between the dietary factors and PCOS in future.
